# Chronic urticaria patients are interested in apps to monitor their disease activity and control: A UCARE CURICT analysis

**DOI:** 10.1002/clt2.12089

**Published:** 2021-12-18

**Authors:** Ivan Cherrez‐Ojeda, Emanuel Vanegas, Annia Cherrez, Miguel Felix, Karsten Weller, Markus Magerl, Rasmus Robin Maurer, Valeria L. Mata, Alicja Kasperska‐Zajac, Agnieszka Sikora, Daria Fomina, Elena Kovalkova, Kiran Godse, Nimmagadda Dheeraj Rao, Maryam Khoshkhui, Sahar Rastgoo, Roberta F. J. Criado, Mohamed Abuzakouk, Deepa Grandon, Martijn B. A. Van Doorn, Solange Oliveira Rodrigues Valle, Eduardo Magalhães De Souza Lima, Simon Francis Thomsen, German D. Ramón, Edgar E. Matos Benavides, Andrea Bauer, Ana M. Giménez‐Arnau, Emek Kocatürk, Carole Guillet, Jose Ignacio Larco, Zuo‐Tao Zhao, Michael Makris, Carla Ritchie, Paraskevi Xepapadaki, Luis Felipe Ensina, Sofia Cherrez, Marcus Maurer

**Affiliations:** ^1^ Universidad Espíritu Santo Samborondón Ecuador; ^2^ Respiralab Respiralab Research Group Guayaquil Ecuador; ^3^ Clinic and Policlinic for Dermatology and Venereology University Medical Center Rostock Rostock Germany; ^4^ Institute for Allergology, Charité ‐ Universitätsmedizin Berlin, corporate member of Freie Universität Berlin and Humboldt‐Universität zu Berlin Berlin Germany; ^5^ Fraunhofer Institute for Translational Medicine and Pharmacology (ITMP), Allergology and Immunology Berlin Germany; ^6^ European Center for Diagnosis and Treatment of Urticaria Medical University of Silesia Katowice Poland; ^7^ City Center of Allergy and Immunology Clinical City Hospital #52 Moscow Russia; ^8^ Department of Allergology and Clinical Immunology I.M. Sechenov First Moscow State Medical University Moscow Russian Federation; ^9^ Department of Dermatology D Y, Patil University School of Medicine and Hospital Mumbai India; ^10^ Allergy Research Center Mashhad University of Medical Sciences Mashhad Iran; ^11^ Department of Allergy and Immunology Mashhad University of Medical Sciences Mashhad Iran; ^12^ Department of Dermatology Faculdade de Medicina do ABC São Paulo Brazil; ^13^ Allergy and Immunology Department Cleveland Clinic Abu Dhabi Abu Dhabi UAE; ^14^ Department of Dermatology Erasmus MC Rotterdam The Netherlands; ^15^ Department of Internal Medicine Hospital Universitário Clementino Fraga Filho Rio de Janeiro Brazil; ^16^ Faculty of Science and Health of Juiz de Fora ‐ SUPREMA ‐ Minas Gerais Rio de Janeiro Brazil; ^17^ Department of Dermatology Bispebjerg Hospital University of Copenhagen, Biomedical Sciences Copenhagen Denmark; ^18^ Instituto de Alergia e Inmunología del Sur, Bahía Blanca Buenos Aires Argentina; ^19^ Centro de Referencia de Alergia Asma e Inmunología ‐ Instituto Nacional del Niño Lima Peru; ^20^ Department of Dermatology University Allergy Center University Hospital Carl Gustav Carus Technical University Dresden Germany; ^21^ Department of Dermatology Hospital del Mar IMIM, Universitat Autònoma Barcelona Spain; ^22^ Department of Dermatology Koç University School of Medicine Istanbul Turkey; ^23^ Department of Dermatology University Hospital Zurich Zurich Switzerland; ^24^ Allergy Department Clinica San Felipe Lima Peru; ^25^ Department of Dermatology and Venereology Peking University First Hospital Beijing China; ^26^ Allergy Unit 2nd Department of Dermatology and Venereology National and Kapodistrian University of Athens “Attikon” University Hospital Athens Greece; ^27^ Allergy Division Hospital Italiano de Buenos Aires Buenos Aires Argentina; ^28^ Allergy Department 2nd Pediatric Clinic National and Kapodistrian University of Athens Athens Greece; ^29^ Federal University of São Paulo São Paulo Brazil; ^30^ Department of Dermatology SRH Zentralklinikum Suhl Suhl Germany

**Keywords:** apps, chronic inducible urticaria, chronic spontaneous urticaria, chronic urticaria, UCARE

## Abstract

**Background:**

Information/communication technologies such as mobile phone applications (apps) would enable chronic urticaria (CU) patients to self‐evaluate their disease activity and control. Yet, recently Antó et al (2021) reported a global paucity of such apps for patients with CU. In this analysis, we assessed patient interest in using apps to monitor CU disease activity and control using questions from the chronic urticaria information and communication technologies (CURICT) study.

**Methods:**

The methodology for CURICT has been reported. Briefly, a 23‐item questionnaire was completed by 1841 CU patients from 17 UCAREs across 17 countries. Here, we analyzed patient responses to the CURICT questions on the use of apps for urticaria‐related purposes.

**Results:**

As previously published, the majority of respondents had chronic spontaneous urticaria (CSU; 63%; 18% chronic inducible urticaria (CIndU) [CIndu]; 19% with both), were female (70%) and in urban areas (75%). Over half of patients were very/extremely interested in an app to monitor disease activity (51%) and control (53%), while only ∼1/10 were not. Patients with both urticaria types versus those with CSU only (odds ratio [OR], 1.36 [1.03–1.79]) and females versus males (OR [95% CI], 1.47 [1.17–1.85]) were more likely to be very to extremely interested in an app to assess disease control.

**Conclusions:**

Overall, half of the patients with CU were very to extremely interested in using an app to assess their disease activity and control. Development of well‐designed apps, specific to disease types (CSU, CIndU, CSU + CIndU, etc), validated by experts across platforms would help improve the management and possibly outcomes of CU treatment while providing important patient information to be used in future research.

## 1 INTRODUCTION

In the recent Urticaria Centers of Reference and Excellence (UCARE) study, chronic urticaria information and communication technologies (CURICT), we assessed use and interest in information and communications technologies (ICTs) for patients with chronic urticaria (CU).^1^, ^2^ We found that almost all CU patients had access to ICTs and most were using these regularly to obtain health and disease‐related information.^1^ Specifically, most patients with CU were interested in ICTs to receive disease information and to communicate with their physicians and other patients about their urticaria.^2^

Information and communications technologies such as mobile phone applications (apps) would enable patients to (i) self‐evaluate their disease activity, impact, and control, (ii) improve self‐management and (iii) optimize their therapy including allergic comorbidities. Yet, very recently, Antó et al (2021) reported a global paucity of such apps for patients with CU.^3^ To date, the unmet need for suitable apps from the patient's point of view remains unknown.

### 1.1 Objective

Using questions from the CURICT study, we assessed patient interest in using apps to monitor CU disease activity and control.

## 2 METHODS

### 2.1 Study design and population

The scope, methodology, conduct, and other results of the CURICT study have been previously reported.^1^, ^2^ Patients were ≥12 years old and previously diagnosed with chronic spontaneous urticaria (CSU) or chronic inducible urticaria (CIndU) by a physician; those with other dermatological diseases or intellectual disabilities were excluded. A 23‐item questionnaire, designed, evaluated, and reviewed by an expert panel of physicians that collected demographic (age, gender, education level, living area, etc) and clinical information (urticaria type, years with diagnosis, etc.) from each patient. Furthermore, participants were asked to quantify their interest using a scale (not interested, slightly interested, moderately interested, very interested, and extremely interested).^1^, ^2^

Here, we analyzed patient responses to the CURICT questions on the use of apps for urticaria‐related purposes. As previously published, the 23‐item questionnaire^2^ was completed by 1841 CU patients from 17 UCAREs across 17 countries. The majority of respondents had chronic spontaneous urticaria (CSU; 63%; 18% CIndU; 19% with both), were female (70%) and lived in urban areas (75%) with a mean age of 41 years, and ∼4 years of disease duration.^1^, ^2^

### 2.2 Statistical analysis

The agreement scale was dichotomized as “agree‐to‐strongly agree” (agree and strongly agree) and “not agree‐to‐strongly agree” (strongly disagree, disagree and undecided). Similarly, the interest scale was dichotomized as “very‐to‐extremely interested” (very interested and extremely interested) and “not very‐to‐extremely interested” (not interested, slightly interested and moderately interested).

A binomial logistic regression analysis was performed to predict the likelihood of being very to extremely interested or not in app development described above given the effects of age, gender, educational level, region, living area, economy according to the World Bank data, urticaria type and years with diagnosis. All data were analyzed using SPSS version 24.0 software (SPSS Inc.).

### 2.3 Ethical considerations

This study was approved by the ethics committee “Comité de ética e Investigación en Seres Humanos” (CEISH), Guayaquil, Ecuador (IRB number HCK‐CEISH‐19‐0059) and by a committee for each participating UCARE center. Each participant provided consent to completion of the anonymous survey, and confidentiality was maintained throughout the study.

## 3 RESULTS

### 3.1 More than half of patients with CU are very to extremely interested in an app to monitor disease activity and control

Over half of patients were very or extremely interested in the development of an app to monitor disease activity (51%; *n* = 946) and control (53%; *n* = 967), while only ∼1/10 were not interested at all in apps to assess such parameters (urticaria activity, 12.8%, *n* = 236; urticaria control, 12.0%, *n* = 221).

### 3.2 Comorbid CIndU in patients with CSU is linked to higher levels of interest in the use of an app to assess disease control

Patients with both urticaria types reported a greater likelihood of being very to extremely interested in app development to assess disease control (odds ratio [OR], 1.36 [1.03–1.79]) versus those with CSU only.

### 3.3 Female patients and those with higher education are significantly more likely to use apps for disease control

Female patients were more likely than males to be very/extremely interested in the development of an app to monitor disease control (Table 1). The two highest levels of education (undergraduate and postgraduate studies) presented the highest odds of being very to extremely interested in the use of an app to assess disease activity (OR [95% CI], 3.60 [2.37–5.48] and 2.56 [1.64–3.99], respectively) and control (OR, 2.82 [1.88–4.23] and 2.36 [1.53–3.64], respectively).

**TABLE 1:** Adjusted logistic regression reporting patient interest in app development to assess urticaria activity and control

	Disease activity	Disease control
Variable	Very to extremely interested in app development	Very to extremely interested in app development
	OR (95% CI)	OR (95% CI)
**Age**	**0.97 (0.96–0.98)**	**0.97 (0.96–0.98)**
Gender^a^		
Female	1.24 (0.98–1.56)	**1.47 (1.17–1.85)**
Education level^b^		
Secondary/Highschool	**2.33 (1.53–3.53)**	**1.75 (1.17–2.62)**
Undergraduate/college	**3.60 (2.37–5.48)**	**2.82 (1.88–4.23)**
Postgraduate studies	**2.56 (1.64–3.99)**	**2.36 (1.53–3.64)**
Economy^c^		
Upper middle income	**0.52 (0.38–0.73)**	**0.47 (0.34–0.66)**
Urticaria type^d^		
Both (CSU & CIndU)	1.26 (0.95–1.67)	**1.36 (1.03–1.79)**

*Note*: Regression analyses were adjusted for variables such as age, gender, education level, region, living area, economy, urticaria type and years with urticaria. Bolded values are significant at 0.05 significance level.

Abbreviations: CI, confidence interval; CIndU, chronic inducible urticaria; CSU, chronic spontaneous urticaria; OR, odds ratio

^a^ Reference gender category is “female”.

^b^ Reference education level category is “No education/Primary school”.

^c^ Reference economy category is “high income”. Categories are defined according to the World Bank data. This classification changes the thresholds to define each category based on the gross national income per capita of each country, which is the dollar value of a country's final income year divided by its population. Currently the thresholds are: Low income (<1046), Lower‐middle income (1046‐4095), Upper‐middle income (4096‐12,625), High income (>12,535).

^d^ Reference urticaria type is CSU.

## 4 OLDER AGE WAS IDENTIFIED AS A SIGNIFICANT PREDICTOR FOR LESS INTEREST IN APP DEVELOPMENT FOR DISEASE ACTIVITY AND CONTROL

Even though older age was identified as a significant predictor for less interest in app development for disease activity and control, the ORs approached but did not exceed the unit value of 1.0 (OR, 0.97 [0.96–0.98]; 0.97 [0.96–0.98], respectively). Furthermore, patients residing in upper middle‐income countries were less likely to be interested in app development to measure disease outcomes versus those in high income countries.

## 5 DISCUSSION

In this study, across all strata, patients with CU demonstrated very/extreme interest in using an app for monitoring their disease activity and control. Being female, higher education, and having both CSU and CIndU were important drivers of this interest.

Other recent studies assessing app use in chronic allergic conditions have demonstrated high patient interest/satisfaction as well compliance and subjective improvements in their disease across age groups.^4^, ^5^, ^6^ Glattacker et al found that adults patients with allergic rhinitis were adherent with over half using the app for >6 months. Patients also reported subjective improvements due to app use including greater knowledge of their allergies, improved QoL, and greater allergy self‐management.^4^ In a study assessing the impact of ASTHMAXcell Adventures, a gamified, guideline‐based, pediatric app, Hsia et al, found that children were highly satisfied with an app and that its use improved asthma control, knowledge, and QoL while reducing emergency department visit and prednisone use.^5^ In young adults with asthma, Murphy et al discovered that use of an app may support adherence in inhaled corticosteroid use.^6^ Moreover, Bousquet et al, were able to identify phenotype differences between *a priori*‐defined rhinitis groups using the MASK‐rhinitis app.^7^ This suggests that research questions for chronic diseases may be better and more easily identified using app technology.

Given our findings, there is a clear unmet need for well‐developed apps, particular for CU disease type, that could comprehensively document patients' daily disease activity (triggers, QoL, comorbidities, consequences) and accurately account for disease control or lack thereof. Apps could also provide detailed and unique data about each patient, which would enable practitioners to develop and implement individualized treatment plans and adapt quickly according to disease course. Yet, while most CU patients are very interested in using apps to document their condition, suitable ones which would thoroughly assess their condition are limited in number, function, geographical reach, and none covered comorbidities; importantly only one app noted by Anto et al assessed disease control.^3^

Our study included a large sample of patients and utilized a network of researchers across the world. However, the study design was cross‐sectional and therefore cause‐and‐effect relationships could not be detected; in addition, while the questionnaire was developed by experts worldwide, it has not been confirmed in validation studies to date. Also, we did not record the disease severity and control, and as such, we cannot identify if these variables could affect the outcomes of interest.

## 6 CONCLUSIONS

Overall, over half of patients with CU were very to extremely interested in using an app to assess their disease activity and control. Development of well‐designed apps, specific to disease types (CSU, CIndU, CSU + CIndU, etc), validated by experts across platforms would help improve the management and possibly outcomes of CU treatment while providing important patient information to be used in future research.

## CONFLICT OF INTEREST

**Ivan Cherrez‐Ojeda** has no conflicts of interest. **Emanuel Vanegas** has no conflicts of interest. **Annia Cherrez** has no conflicts of interest. **Miguel Felix** has no conflicts of interest. **Karsten Weller** is or recently was a speaker and/or advisor for, and/or has received research funding from: Biocryst, CSL Behring, Dr. Pfleger, FAES, Moxie, Novartis, Shire/Takeda, and Uriach. **Markus Magerl** is or recently was a speaker and/or advisor for, and/or has received research funding from Biocryst, CSL Behring, Kalvista Pharmaceuticals, Moxie, Novartis, Pharming, and Shire/Takeda. **Rasmus Robin Maurer** has no conflicts of interest. **Valeria L Mata** has no conflicts of interest. **Alicja Kasperska‐Zajac** has no conflicts of interest. **Agnieszka Sikora** has no conflicts of interest. **Daria Fomina** is or recently was a speaker and/or advisor for, and/or has received research funding from: AstraZeneca, CSL Behring, Glaxo SmithKline, MSD, Novartis, Sanofi, and Shire/Takeda. **Elena Kovalkova** has no conflicts of interest. **Kiran Godse** has no conflicts of interest. **Nimmagadda Dheeraj Rao** has no conflicts of interest. **Maryam Khoshkhui** has no conflicts of interest. **Sahar Rastgoo** has no conflicts of interest. **Roberta FJ Criado** has no conflicts of interest. **Mohamed Abuzakouk** has no conflicts of interest. **Deepa Grandon** has no conflicts of interest. **Martijn BA van Doorn** is or recently was a speaker and/or advisor for, and/or has received research funding from Abbvie, BMS, Celgene, Janssen Cilag, LEO Pharma, Lilly, MSD, Novartis, Pfizer, and Sanofi‐Genzyme. **Solange Oliveira Rodrigues Valle** has no conflicts of interest. **Eduardo Magalhães de Souza Lima** has no conflicts of interest. **Simon Francis Thomsen** is or recently was a speaker and/or advisor for, and/or has received research funding from: Abbvie, AstraZeneca, Celgene, Eli Lilly, Janssen, LEO Pharma, Novartis, Pierre Fabre, Roche, Sanofi, and UCB. **German D Ramón** has no conflicts of interest. **Edgar E Matos Benavides** has no conflicts of interest. **Andrea Bauer** is or recently was a speaker and/or advisor for, and/or has received research funding from Novartis, Sanofi, Genentech, Leo Pharma and Shire/Takeda. **Ana M Giménez‐Arnau** has held roles as a Medical Advisor for Sanofi, Uriach, Genentech, Novartis, Amgen, ThemoFisher, Roche, Almirall, and has research grants supported by Instituto Carlos III‐ FEDER, Novartis, and Uriach; she also participates in educational activities for Almirall, Genentech, Glaxo SmithKline, LEO Pharma, Menarini, MSD, Novartis, Sanofi, Avene and Uriach. **Emek Kocatürk** is or recently was a speaker and/or advisor for Bayer, Novartis, and Sanofi. **Carole Guillet** has no conflicts of interest. **Jose Ignacio Larco** is or recently was a speaker and/or advisor for: FAES, Novartis, and Sanofi. **Zuo‐Tao Zhao** has no conflicts of interest. **Michael Makris** is or recently was a speaker and/or advisor for, and/or has received research funding from AstraZeneca, Chiesi, Glaxo SmithKline, Novartis, and Sanofi. **Carla Ritchie** has no conflicts of interest. **Paraskevi Xepapadak** reports personal fees from Galenica Greece, Glaxo SmithKline, Nestle, Novartis, Nutricia, and Uriach, outside the submitted work. **Luis Felipe Ensina** is or recently was a speaker and/or advisor for, and/or has received research funding from Novartis, Sanofi, and Takeda. **Sofia Cherrez** has no conflicts of interest. **Marcus Maurer** is or recently was a speaker and/or advisor for, and/or has received research funding from: Allakos, Alnylam, Aralez, AstraZeneca, Biocryst, Blueprint, CSL Behring, FAES, Genentech, Kalvista Pharmaceuticals, LEO Pharma, Menarini, Moxie, MSD, Novartis, Pharming, Pharvaris, Roche, Sanofi, Shire/Takeda, UCB, and Uriach.
